# Effect of Liraglutide on Cardiometabolic Risk Profile in People with Coronary Artery Disease with or without Type 2 Diabetes: A Systematic Review and Meta-Analysis of Randomized Controlled Trials

**DOI:** 10.3389/fphar.2021.618208

**Published:** 2021-03-29

**Authors:** Peyman Nowrouzi-Sohrabi, Negin Soroush, Reza Tabrizi, Mojtaba Shabani-Borujeni, Shahla Rezaei, Fatemeh Jafari, Mahnaz Hosseini-Bensenjan, Bruno H. Stricker, Mandy van Hoek, Fariba Ahmadizar

**Affiliations:** ^1^ Department of Biochemistry, School of Medicine, Shiraz University of Medical Sciences, Shiraz, Iran; ^2^ Student Research Committee, Shiraz University of Medical Sciences, Shiraz, Iran; ^3^ Department of Epidemiology, Erasmus Medical Center, Rotterdam, Netherlands; ^4^ Noncommunicable Diseases Research Center, Fasa University of Medical Sciences, Fasa, Iran; ^5^ Health Policy Research Center, Institute of Health, Shiraz University of Medical Sciences, Shiraz, Iran; ^6^ Department of Clinical Pharmacy, Faculty of Pharmacy, Shiraz University of Medical Sciences, Shiraz, Iran; ^7^ Nutrition Research Center, School of Nutrition and Food Sciences, Shiraz University of Medical Sciences, Shiraz, Iran; ^8^ Hematology Research Center, Shiraz University of Medical Sciences, Shiraz, Iran; ^9^ Department of Internal Medicine, Erasmus Medical Center, Rotterdam, Netherlands

**Keywords:** liraglutide, cardiometabolic profiles, coronary artery disease, systematic review, meta-analysis

## Abstract

**Background**: Whether liraglutide use improves cardiometabolic risk factors in different subsets of subjects with coronary artery disease (CAD) remains unclear. In a systematic review and meta-analysis, we quantified the effects of liraglutide on cardiometabolic risk profile in subjects with CAD with or without type 2 diabetes mellitus (T2D).

**Methods**: Online database searches were conducted in PubMed, Scopus, EMBASE, Web of Science, Cochrane library, and Google Scholar from incept up to 15th January 2021. We identified randomized controlled trials (RCTs) assessing the effects of liraglutide compared to placebo on cardiometabolic risk profile. We used the random- or fixed-effect models to pool the weighted mean differences (WMDs) and 95% confidence intervals (CIs).

**Results**: Out of a total of 7,320 citations, six articles (seven RCTs) with 294 subjects with CAD (mean age, 61.21 years; 19% women) were included. Our findings presented as WMD and 95% CI showed a statistical significant decrease in hemoglobin A1c (HbA1c) [−0.36%; −0.47; −0.26, *p* < 0.001; *I* ^2^ = 0.0% (with 6 RCTs)], body mass index (BMI) [−0.61 kg/m^2^; −1.21; −0.01, *p* = 0.047; *I* ^2^ = 72.2% (with five RCTs)], and waist circumference [−2.41 cm; −3.47; −1.36, *p* < 0.001; *I* ^2^ = 0.0% (with three RCTs)]. Through a set of subgroup analyses, we found a significant reduction in BMI in CAD patients with T2D [WMD = −1.06; 95% CI, −1.42, −0.70, *p* < 0.001; *I* ^2^ = 0.0% (with three RCTs)] compared to CAD only patients [WMD = −0.08; 95% CI, −0.45, 0.29, *p* = 0.66; *I* ^2^ = 0.0% (with two RCTs)] in the liraglutide group compared with the placebo group. No significant changes in heart rate, blood pressure, and lipid profiles were observed.

**Conclusions**: Among people with established CAD, liraglutide significantly improved HbA1c, BMI, and waist circumference values. The effect of liraglutide on BMI was more robust in individuals with T2D compared to those without.

## Introduction

Liraglutide is an analog of human native incretin hormone with 97% similarity and is known as a long-acting glucagon-like peptide 1 receptor agonist (GLP1-RA) (Howell et al., 2019). Liraglutide is used as a dual therapy option after first-line metformin therapy in type 2 diabetes mellitus (T2D)patients with established CVD (Cosentino et al., 2019; Buse et al., 2020). Liraglutide can also be considered inT2D patients aged 55 years or older and high risk of CVD, even without established atherosclerotic cardiovascular disease (ASCVD), to reduce the risk of major adverse cardiovascular events (MACE) (Buse et al., 2020). The results of a recent meta-analysis revealed that GLP1-RAs reduce the risk of myocardial infarction (MI), stroke, and cardiovascular death by approximately 14% in T2D patients with known ASCVD (Zelniker et al., 2019). The multifaceted mechanism of liraglutide’s action induces increased insulin secretion, decreased glucagon secretion, and delayed gastric emptying (Howell et al., 2019).

Coronary artery disease (CAD) involves blood flow impairment through the coronary arteries. Silent ischemia and angina pectoris are among the most common CAD clinical presentation. Most importantly, CAD is a predominant risk factor for sudden cardiac death and heart failure progression (Velagaleti & Vasan, 2007; Malavolta et al., 2017). Optimal treatment of CAD leads to improve patients’ survival rates for many years and decrease the disease progression and complications (Malavolta et al., 2017).

Liraglutide has favorable effects on cardiometabolic risk factors in T2D patients (Davies et al., 2015; Sun et al., 2015; Sun et al., 2015; Liakos et al., 2019; Matikainen et al., 2019). Whether this medication improves cardiometabolic risk profiles in CAD patients remains unclear. Therefore, we conducted a systematic review and meta-analysis of randomized controlled trials (RCT) to assess and quantify the effect of liraglutide on cardiometabolic traits including glycemic traits, body mass index (BMI), waist circumference (WC), blood pressure, heart rate, and lipids traits in patients with established CAD. Whether coincidence of CAD with T2D can influence the effect of liraglutide on cardiometabolic risk profile was also evaluated.

## Materials and Methods

The current systematic review and meta-analysis was performed and reported according to the items in the Preferred Reporting Items for Systematic Reviews and Meta-analyses (PRISMA) guidelines.

### Search Strategy

Online database searches were conducted in PubMed, Scopus, EMBASE, Web of Science, the Cochrane library, and an additional search in Google Scholar from inception to the 15th of January 2021. To increase our searches’ sensitivity, we manually checked the reference lists of relevant studies and previous reviews. The search strategy was performed using the following pattern: (Key terms for liraglutide)AND (Key terms for population/ interested outcomes) AND (Key terms for study design). Scopus search trips as an example are provided in Supplementary Appendix 1.

### Inclusion and Exclusion Criteria

Two authors (MSh-B and FJ) independently evaluated all retrieved citations using the inclusion criteria. Discrepancies were resolved through consensus or discussion with a third author (RT or PN-S). We included all published RCTs in English (either with parallel or cross-over design) that investigated the effect of liraglutide use on cardiometabolic profile in CAD patients with or without T2D. No date limitations were on the studies’ identification.

### Data Extraction

Two independent authors (MSh-B and FJ) extracted the following information from the selected studies: first author, year of publication, study design, the mean age of participants, study population, mean (SD) changes of cardiometabolic traits including hemoglobin A1c (HbA1c), BMI, WC, systolic blood pressure (SBP), diastolic blood pressure (DBP), triglycerides (TG), total cholesterol (TC), low-density lipoprotein (LDL)-cholesterol, and high-density lipoprotein (HDL)-cholesterol), the number of participants in total and each group (intervention and control), type of intervention, type of placebo, and duration of intervention. In case of disagreement between the two authors, the consensus was reached by a third author (F.A).

### Quality Assessment

**FIGURE 1:**
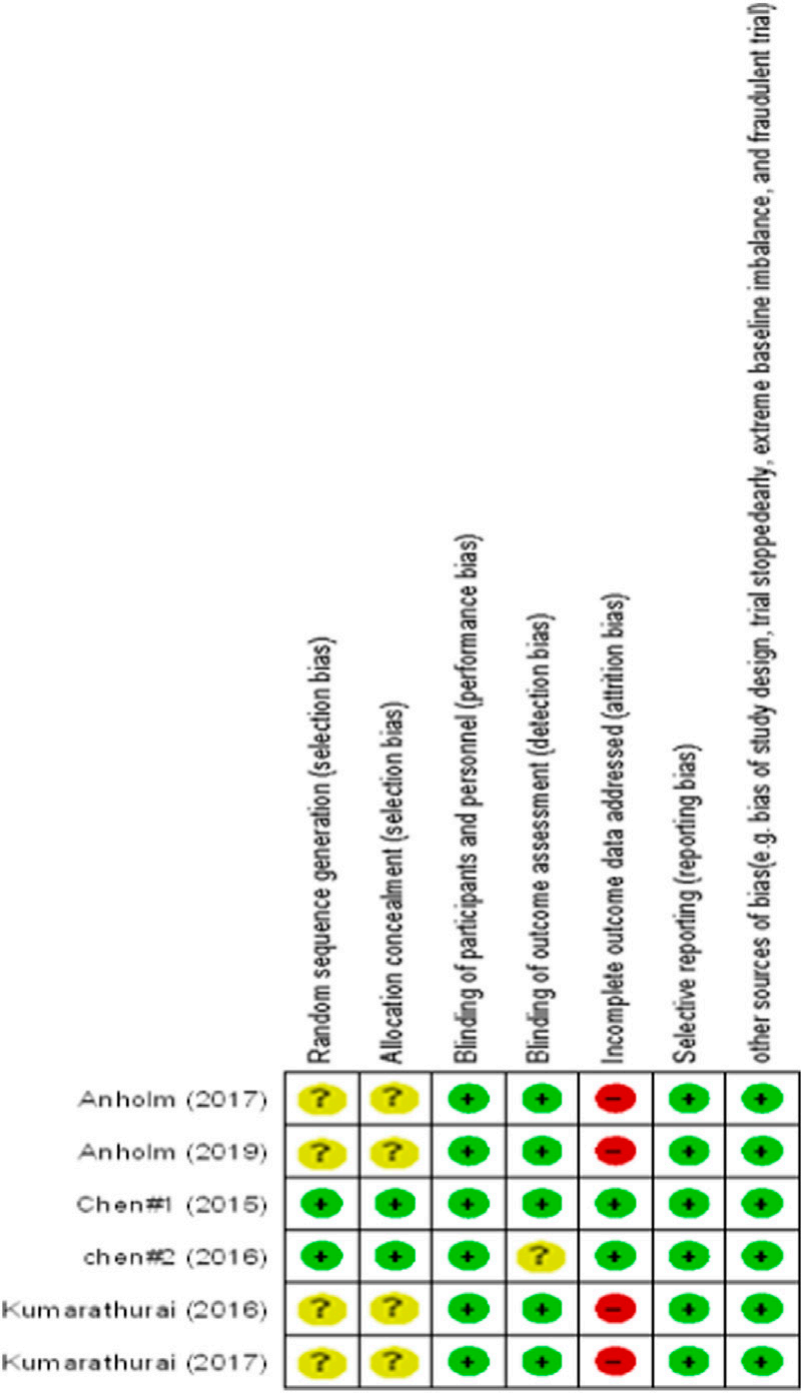
Quality assessment of included studies.

The quality of the selected studies was critically assessed using the Cochrane Collaboration Risk of Bias tool. The quality items included “randomization generation, allocation concealment, blinding of subjects and outcome assessment, incomplete outcome data, selective outcome reporting, and other sources of bias.” The results of the quality assessment of included studies are presented in Figure 1.

### Statistical Analysis

All statistical analyses were performed using STATA version 12.0 (Stata Corp., College Station, TX) and RevMan software (Cochrane Review Manager, version 5.2). The effect of liraglutide on cardiometabolic traits was reported as mean (SD) change in the intervention and the placebo groups. Once included RCTc did not report mean (SD) change, we calculated the mean changes, and their corresponding SDs using the following formula: [mean_post_ − mean_pre_] and [√ ([SD_pre_^2^ + SD_post_^2^] – [2 × *R* × SD_pre_ × SD_post_])] (Borenstein et al., 2011), respectively. The correlation coefficient (*R*) was calculated based on the study conducted by Kumarathurai et al. (2017) using an appropriate formula proposed by Cochrane guidelines for systematic reviews and meta-analysis (Higgins, 2011). In Chen et al. (2016) study, CIs for mean changes for each group (intervention/control) were used to calculate their SDs by the following formula: [SD = √*N* × (upper limit – lower limit)/2 × a value from a t distribution]; t value for a 95% CI from a sample size of 45 subjects (per each group) was obtained as 2.02. Statistical heterogeneity across selected trials was determined using Cochrane’s Q test and the *I* ^2^ statistic with *I* ^2^ > 50% and Cochrane’s Q test as *p* < 0.1, indicating the existence of significant heterogeneity across included studies. We used the random-effects model [with DerSimonian–Laird method] to pool the weighted mean differences (WMDs) and 95% confidence intervals (CIs); otherwise, the fixed-effect model [with inverse variance method] was applied. In a set of subgroup analyses, we assessed liraglutide’s effect on the cardiometabolic risk profile, in patients with CAD comparing those with and without T2D. To assess our findings’ robustness, a series of sensitivity analyses were conducted with the leave-one-out method to assess the impact of each included study on the pooled WMDs. We assessed the presence of potential evidence of publication bias using the Egger regression and Begg’s rank correlation tests, with *p* < 0.05 suggesting publication bias.

## Results

**FIGURE 2:**
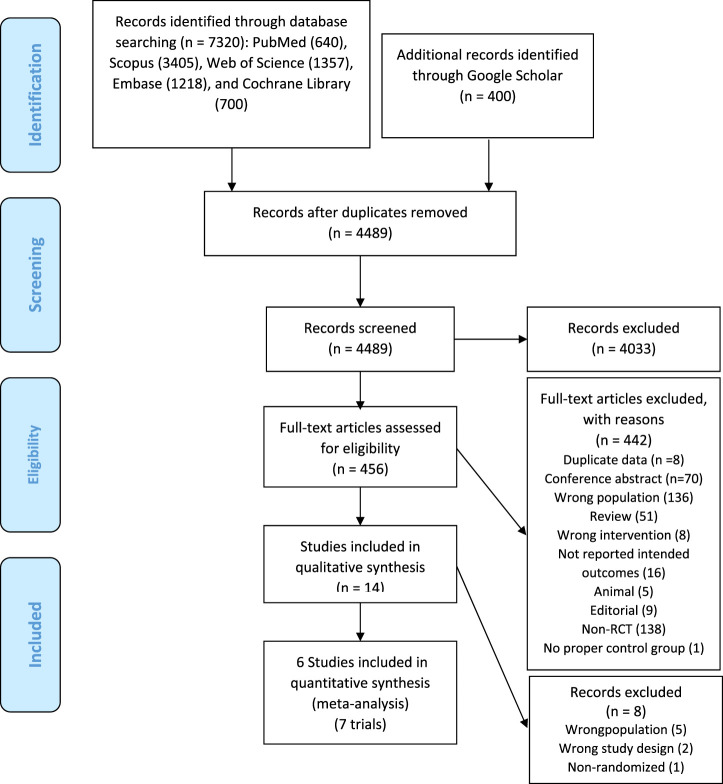
Flowchart of the identification studies and selection process.

A total of 7,320 citations were identified through electronic database searches. Of these, 3,231 were duplicate reports. After screening titles and abstracts, 456 full texts were retrieved for assessment. Finally, six articles (including seven RCTs, as some articles contained more than one study) (Chen et al., 2015; Chen et al., 2016; Kumarathurai et al., 2016; Anholm et al., 2017; Kumarathurai et al., 2017; Anholm et al., 2019) were included in our meta-analysis. The process of study identification and selection is presented in Figure 2.

The design of four included studies was cross-over (Kumarathurai et al., 2016; Anholm et al., 2017; Kumarathurai et al., 2017; Anholm et al., 2019) while two had a parallel design (Chen et al., 2015; Chen et al., 2016). Six RCTs had reported data for calculating changes in HbA1c; five had data on BMI, SPB, DBP, four on heart rate, three on WC, LDL-cholesterol, and two on TG, TC, HDL-cholesterol. The characteristics of RCTs included in the meta-analysis are summarized in Table 1.

**TABLE 1:** Characteristics of included studies.

Authors (ref)	Publication year	Sample size (control/intervention)	Country/population	Intervention group	Duration of treartment	Duration of follow-up	Study design	Age (control, intervention)	Presented data
Chen et al. (2015)	2015	7/9	China/DM acute ST-segment elevation myocardial infarction (STEMI) undergoing primary PCI	Liraglutide (0.6 mg once daily for 2 days (1.6 pmol/kg per minute), 1.2 mg for another 2 days (3.2 pmol/kg per minute), and 1.8 mg for 3 days (4.8 pmol/kg per minute))	1 week^a^	12 weeks	Single-center, randomized, double-blind, placebo, controlled trial	59.2 ± 14.4 57.7 ± 11.3	BMI, HbA1C, SBP, DBP, and HR
Chen et al. (2015)	2015	40/36	China/NDM acute ST-segment elevation myocardial infarction (STEMI) undergoing primary PCI	Liraglutide (0.6 mg once daily for 2 days (1.6 pmol/kg per minute), 1.2 mg for another 2 days (3.2 pmol/kg per minute), and 1.8 mg for 3 days (4.8 pmol/kg per minute))	1 week^a^	12 weeks	Single-center, randomized, double-blind, placebo, controlled trial	59.2 ± 14.4 57.7 ± 11.3	BMI, HbA1C, SBP, DBP, and HR
Chen et al. (2016)	2016	45/45	China/Non-STsegment elevation myocardial infarction (NSTEMI)	Liraglutide (0.6 mg once daily for 2 days, 1.2 mg liraglutide for another 2 days, followed by 1.8 mg liraglutide for 3 days)injection	1 week^a^	12 weeks	Single-center, randomized, double-blind, placebo, controlled trial	59.0 ± 12.1 58.0 ± 11.7	BMI, HbA1C, SBP, DBP, HR, TG, TC, HDL-C, and LDL-C
Kumarathurai et al. (2016)	2016	30	Denmark/Patients with CAD and T2D	0.6 mg liraglutide (injection) od + 500 mg metformin (tablet) bid was increased after 14 days to 1.2 mg od + (1,000 mg + 500 mg) daily and to 1.8 mg od + 1,000 mg bid after 28 days	12 weeks	12 weeks	Randomized, double-blind, placebo-controlled 12 plus 12 weeks crossover study	61.8 ± 7.6	BMI, WC, HbA1C, SBP, DBP, HR, and LDL-C
Kumarathurai et al. (2017)	2017	24	Denmark/Overweight patients with newly diagnosed T2D and stable CAD	0.6 mg liraglutide once daily (o.d.) + 500 mg metformin twice daily (b.i.d.) was increased after 14 days to liraglutide 1.2 mg o.d.+ metformin (1000 mgþ500 mg) and to 1.8 mg o.d. + 1000 mg **B**.i.d. after 28 days	12 weeks	12 weeks	Randomized, double-blind, placebo-controlled, crossover study	62.5 + 7.2	WC, HbA1C, SBP, and DBP
Anholm et al. (2017)	2017	30	Denmark/Overweight patients with CAD and newly diagnosed T2D	Liraglutide (1.8 mg once daily (titrated from 0.6 to 1.8 mg during 4 weeks)) + metformin (1 g twice daily (titrated from 500 mg to 1 g during 4 weeks))	12 weeks	12 weeks	Investigator-initiated, double-blinded, randomized, placebo-controlled, crossover trial	62.3 ± 7.6	BMI, WC, and HbA1C
Anholm et al. (2019)	2019	28	Denmark/Patients with CAD and newly diagnosed T2D	Liraglutide once daily was titrated from 0.6 to 1.8 mg within 4 weeks and metformin was titrated from 500 mg twice daily to 1 g twice daily in 4 weeks	12 weeks	12 weeks	Investigator-initiated, double-blind, randomized, placebo-controlled, cross-over trial	62.3 ± 7.6	TG, TC, HDL-C, and LDL-C

Abbreviations: CAD, coronary artery disease; Non-DM, Non-diabetes mellitus; DM, diabetes mellitus; T2D, type 2 diabetes; PCI, percutaneous coronary intervention; BMI, body mass index; WC, waist circumference; HR, heart rate; HDL-C, high density lipoprotein-cholesterol; LDL-C, low density lipoprotein-cholesterol; TG, triglycerides; TC, total cholesterol; HbA1C, hemoglobin A1C; SBP, systolic blood pressure; DBP, diastolic blood pressure.

^a^ Duration of the follow-up was 12 weeks.

### The Effects of Liraglutide Use on Cardiometabolic Traits

**FIGURE 3:**
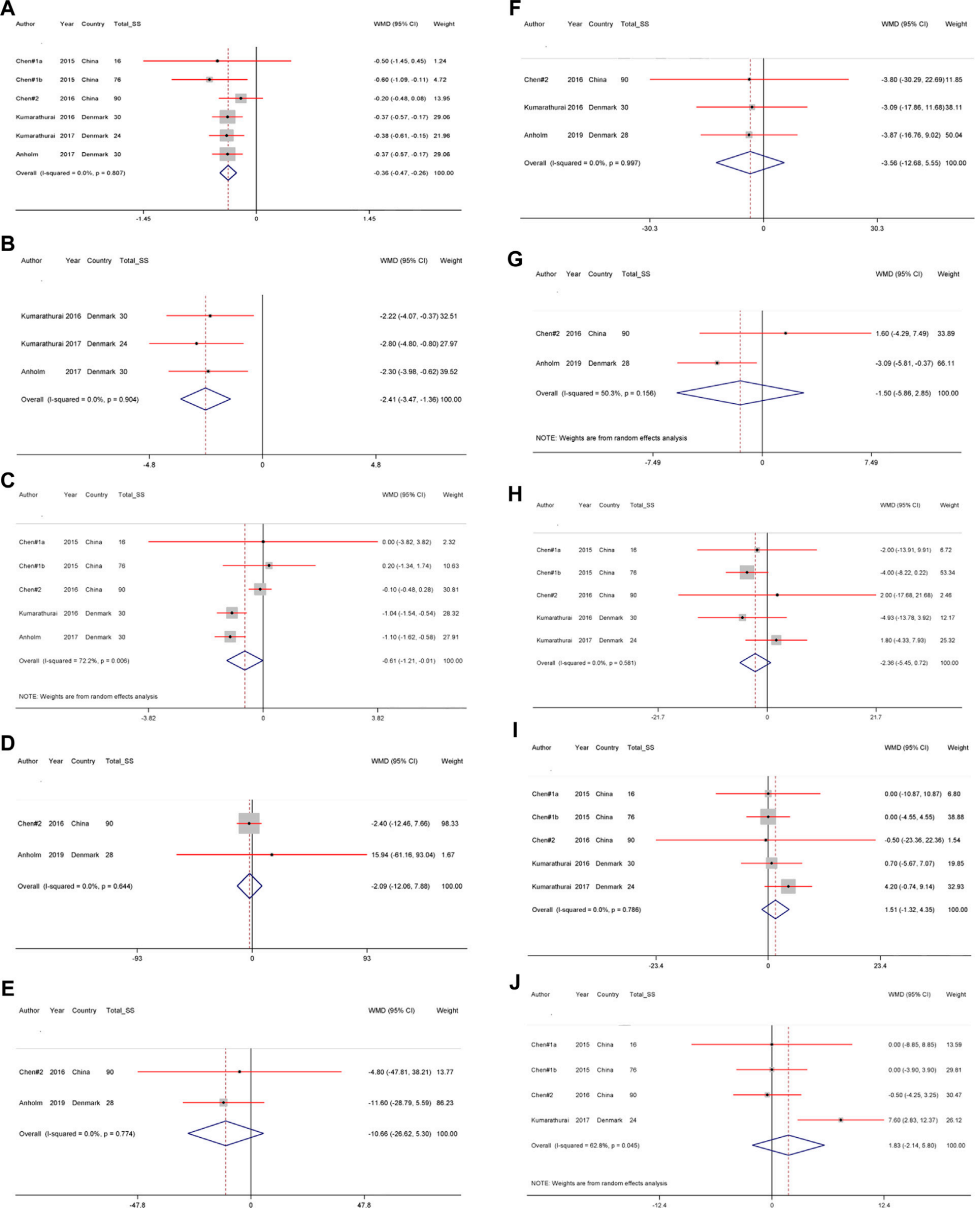
**A)–(J)** The effect of liraglutide use on **(A)** hemoglobin A1c, **(B)** body mass index **(C)** waist circumference, **(D)** triglycerides **(E)** total-cholesterol, **(F)** low-density lipoprotein–cholesterol **(G)** high-density lipoprotein–cholesterol, **(H)** systolic blood pressure, **(I)** diastolic blood pressure and **(J)** Heart rate levels.

The effects of liraglutide on cardiometabolic traits among patients with CAD are shown in Figures 3A–M.

Using fixed-effect model, our meta-analyses showed a significant decrease in the WMD of HbA1c [WMD = −0.36%; 95% CI, −0.47; −0.26, *p* < 0.001; *I* ^2^ = 0.0% (with six RCTs)], and WC [WMD = −2.41 cm; 95% CI, -3.47; - 1.36, *p* < 0.001; *I* ^2^ = 0.0% (with three RCTs)], and according to random-effects model, a significant decrease in the WMD of BMI [WMD = −0.61 kg/m^2^; 95% CI, −1.21; −0.01, *p* = 0.047; *I* ^2^ = 72.2% (with five RCTs)] in the liraglutide group compared with the placebo group.

Liraglutide had no significant effect on lipid traits, including TG [WMD = −2.09 mg/dl; 95% CI, −12.06; 7.88, *p* = 0.681; *I* ^2^ = 0.0% (with two RCTs)], TC [WMD = −10.66 mg/dl; 95% CI, −26.62; 5.30, *p* = 0.190; *I* ^2^ = 0.0% (with two RCTs)], LDL-cholesterol [WMD = −3.56 mg/dl; 95% CI, −12.68; 5.55, *p* = 0.444; *I* ^2^ = 0.0% (with three RCTs)], and HDL-cholesterol [WMD = −1.50 mg/dl; 95% CI, −5.86; 2.85, *p* = 0.499; *I* ^2^ = 50.3% (with two RCTs)].

No significant changes in SBP [WMD = −2.36%; 95% CI, −5.45; 0.72, *p =* 0.133; *I* ^2^ = 0.0% (with five RCTs)], DBP [WMD = 1.51%; 95% CI, −1.32; 4.35, *p* = 0.295; *I* ^2^ = 0.0% (with five RCTs)], and heart rate [WMD = 1.83 bpm (beat per minute); 95% CI, −2.14; 5.80, *p* = 0.366; *I* ^2^ = 62.8% (with four RCTs)] were observed.

### Subgroup Analysis

As shown in Supplementary Table S1, Liraglutide effects in CAD patients with and without T2D were analyzed and compared through a set of subgroup analyses. We found a significant difference between the two groups in whichCAD patients with T2D had significantly more reduction in BMI [WMD = −1.06 kg/m^2^; 95% CI, v1.42; −0.70, *p* < 0.001; *I* ^2^ = 0.0% (with three RCTs)] compared to CAD only patients [WMD = −0.08 kg/m^2^; 95% CI, −0.45; 0.29, *p* = 0.660; *I* ^2^ = 0.0% (with two RCTs)]. In CAD patients with T2D, HDL-cholesterol decreased significantly compared to their comparator. No significant differences between CAD patients with and without T2D have been observed for the effect of liraglutide on the other cardiometabolic traits.

### Sensitivity Analysis

Our sensitivity analyses showed no significant differences between the pre-and post-sensitivity WMDs after excluding each included RCT for HbA1c, WC, DBP, heart rate, and LDL-cholesterol. However, there was a significant change in the pooled WMD of the BMI and SBP in the liraglutide compared to the placebo group after removing Anholm et al. study (WMD = −0.41 kg/m^2^; 95% CI, −1.14; 0.30) (Anholm et al., 2017) and Kumarathurai et al. study (WMD = −3.77%; 95% CI, −7.34; −0.20) (Kumarathurai et al., 2017) , respectively (Table 2).

**TABLE 2:** The effect of one by one trial in the association between liraglutide use and cardiometabolic profiles using sensitivity analysis.

Variable	Pre-sensitivity analysis			Upper and lower of effect size	Post-sensitivity analysis		
	No. of studies included	Pooled WMD	95% CI		Pooled WMD	95% CI	Excluded studies
HbA1C	6	−0.36	−0.47, −0.26	Upper	−0.34	−0.45, −0.24	Chen et al. (2015)
				Lower	−0.38	−0.50, −0.27	Chen et al. (2016)
BMI	5	−0.61	−1.21, −0.01	Upper	−0.41	−1.14, 0.30	Anholm et al. (2017)
				Lower	−0.99	−1.34, −0.64	Chen et al. (2016)
WC	3	−2.41	−3.47, −1.36	Upper	−2.26	−3.50, −1.01	Kumarathurai et al. (2017)
				Lower	−2.50	−3.79, −1.22	Kumarathurai et al. (2016)
SBP	5	−2.36	−5.45, 0.72	Upper	−0.49	−5.00, 4.02	Chen et al. (2015)
				Lower	−3.77	−7.34, −0.20	Kumarathurai et al. (2017)
DBP	5	1.51	−1.32, 4.35	Upper	2.47	−1.15, 6.10	Chen et al. (2015)
				Lower	0.19	−3.26, 3.65	Kumarathurai et al. (2017)
Heart rate	4	1.83	−2.14, 5.80	Upper	2.81	−2.74, 8.37	Chen et al. (2016)
				Lower	−0.23	−2.82, 2.34	Kumarathurai et al. (2017)
LDL-cholesterol	3	−3.56	−12.68, 5.55	Upper	−3.26	−16.16, 9.63	Anholm et al. (2019)
				Lower	−3.85	−15.44, 7.73	Kumarathurai et al. (2016)

Abbreviations: HbA1c, hemoglobin A1c; BMI, body mass index; WC, waist circumference; SBP, systolic blood pressure; DBP, diastolic blood pressure; HR, heart rate; TG, triglycerides; TC, total cholesterol; HDL, high-density lipoprotein; LDL, low-density lipoprotein-cholesterol.

### Publication Bias

Egger regression and Begg’s rank correlation tests indicated no significant evidence of potential publication bias for cardiometabolic traits including, HbA1C (*P*_Eg_ = 0.53, *P*_Be_ = 0.26), BMI (*P*_Eg_ = 0.97, *P*_Be_ = 0.62), WC (*P*_Eg_ = 0.47, *P*_Be_ = 0.60), SBP (*P*_Eg_ = 0.63, *P*_Be_ = 0.33), DBP (*P*_Eg_ = 0.71, *P*_Be_ = 0.62), heart rate (*P*_Eg_ = 0.81, *P*_Be_ = 0.17), and LDL-cholesterol (*P*_Eg_ = 0.98, *P*_Be_ = 0.60). For traits assessed through meta-analysis with lower than three studies, it was impossible to assess the evidence of publication bias.

## Discussion

To our best knowledge, this is the first meta-analysis focused on RCTs that assessed the effect of liraglutide on various cardiometabolic traits in subjects with established CAD. To date, the cardioprotective mechanism of liraglutide has not been elucidated. The suggested conventional explanations are favorable improvements in cardiometabolic risk factors (HbA1c, body weight, SBP, and lipids) or direct action on heart and blood vessels as probable mechanisms (Nauck et al., 2017). Our meta-analysis of seven RCTs included 294 patients with established CAD and revealed that liraglutide treatment significantly decreases HbA1c levels and anthropometric measurements of BMI and WC. We also showed that the positive effect of liraglutide on BMI was more robust in CAD patients with T2D compared to those without.

Since, the CAD is more prevalent among older adults (Rodgers et al., 2019), our target population from the included RCTs, and subsequently study findings were narrowed down to the older population. There is a growing body of evidence suggesting that GLP1-RA may reduce mortality and cardiovascular outcomes, including fatal or non-fatal MI and stroke in T2D patients, beyond their beneficial effect on glycemic control (Kristensen et al., 2019). The LEADER (Liraglutide Effect and Action in Diabetes: Evaluation of Cardiovascular Outcome Results) trial was the major RCT evaluating the efficacy and safety of liraglutide and revealed that T2D patients on liraglutide therapy had 13% lower MACE rates compared to placebo (Marso et al., 2016). The most recent LEADER post hoc analysis proves the efficacy of liraglutide treatment in patients with T2D and high risk of CVD, associated with a reduced risk of first and recurrence MACE (Verma et al., 2019). These cardio-protective effects of liraglutide might be derived from its effects on cardiometabolic traits that eventually influence cardiovascular events risk; however, the exact underlying mechanism remains uncertain. LEADER study may only reveal the potential beneficial effect of liraglutide in patients with T2D at high risk of CVD events, while our study included all RCTs performed in patients with known CAD, regardless of diabetes status.

Concerning the effect of liraglutide on glycemic traits, we found that in patients with liraglutide treatment compared to the placebo group, HbA1c levels decreased by approximately 0.36% with no significant difference between CAD subjects with and without T2D. This finding was concordant with 0.40% reduced HbA1c reported in the previous RCT among T2D patients (Marso et al., 2016).

Our finding showed BMI was reduced by 0.61 kg/m^2^ in patients on liraglutide use compared to the placebo group. Moreover, in a subgroup analysis liraglutide significantly reduced BMI only in CAD patients with T2D. Liraglutide induces weight loss in obese non-diabetic patients by reduction of appetite and energy intake rather than the increase of energy expenditure (van Can et al., 2014). A recent meta-analysis indicates that liraglutide can be considered as an effective and safe treatment for obesity in non-diabetic individuals (Zhang et al., 2019). However, this effect is dose-dependent up to 3.0 mg once daily, with consistent therapy for at least 12 weeks (Zhang et al., 2019). This might explain our observation of a significant reduction in BMI only in CAD patients with T2D. However, we also think that the difference in RCTs’ treatment duration from one week to a maximum of 12 weeks possibly impacted the potential effect on these markers over time*.* Our finding also suggests that liraglutide affects abdominal obesity, as estimated by WC, in which WC measures were reduced by 2.41 cm in the liraglutide group compared to the placebo group. This effect was consistent with a previous study demonstrating the beneficial effect of GLP1-RAs agents on WC in T2D patients, especially in liraglutide users (Sun et al., 2015). It is well known that improvements in abdominal obesity and visceral fat accumulation are associated with reduced insulin resistance and a reduction in major cardiovascular risk factors (Ross and Janiszewski, 2008). Decreased BMI and WC by liraglutide partly explain the beneficial effect of liraglutide on CVD outcomes (Ross and Janiszewski, 2008). Our results showed no significant effect of liraglutide on heart rate, blood pressure, and lipid traits when compared with the placebo effects,which is discordant with previous studies conducted in T2D patients (Sun et al., 2015). These results might be due to the different responses of patients without diabetes to liraglutide treatment by different mechanisms such as attenuation of gastric lipid production or delayed gastric emptying rather than insulin resistance reduction in T2D patients (Hermansen et al., 2013; Madsbad, 2019).

This study has some limitations that should be acknowledged. First, our analysis was based on only six RCTs, including seven studies with a relatively small sample size (*n* < 100) in each trial and short follow-up; thus, caution should be applied while interpreting the results of our study. All of the studies included were performed among patients in two countries of Denmark and China. It is worth mentioning that the process of patient recruitment in two trials by Chen et al. (2015) and Chen et al. (2016) was performed in the hospital settings, while the remaining RCTs used the out-patients data with documented established CAD, according to their inclusion criteria (Kumarathurai et al., 2016; Anholm et al., 2017; Kumarathurai et al., 2017; Anholm et al., 2019). Hospital admitted patients in the acute phase of their CAD conditions might develop worse baseline health measurements and cardiometabolic profiles compared to stable out-patients. Therefore, long-term RCTs with a larger sample size in a more homogeneous target populations, within different ethnic backgrounds should be conducted to confirm our findings. Besides, as it can be argued that the inclusion of patients without diabetes with few major metabolic disorders might reduce the potential effect of liraglutide treatment, we suggest future RCTs in CAD patients with and without diabetes in separated groups.

## Conclusion

Among the population with established coronary artery disease, liraglutide treatment compared with placebo was associated with improved glycemic control and anthropometric measurements, where the effect on BMI was more robust in patients with T2D compared to those without.

## Data Availability

The data that support the findings of this study are available on request from the corresponding or first author.
