# Supplementary material for: Effect of Liraglutide on Cardiometabolic Risk Profile in People with Coronary Artery Disease with or without Type 2 Diabetes: A Systematic Review and Meta-Analysis of Randomized Controlled Trials
Source: Front Pharmacol. 2021 Mar 29;12:618208. doi: 10.3389/fphar.2021.618208 (PMC8039463; doi:10.3389/fphar.2021.618208)
Supplement: Supplementary file 1 [file table1.docx]

**Appendix 1**: Search strategy

**Scopus**

2,846 document results

( INDEXTERMS ( liraglutide OR saxenda OR victoza OR "GLP-1 analog" OR "glucagon-like peptide-1 analog" OR "GLP-1 receptor agonist" OR "glucagon-like peptide-1 receptor agonist" ) OR TITLE-ABS-KEY ( liraglutide OR saxenda OR victoza OR "GLP-1 analog" OR "glucagon-like peptide-1 analog" OR "GLP-1 receptor agonist" OR "glucagon-like peptide-1 receptor agonist" ) ) AND ( INDEXTERMS ( "Coronary Artery Disease" OR "Artery Disease" OR "Coronary Arteriosclerosis" OR "Coronary Atherosclerosis" OR atherosclerosis OR cad OR "Myocardial Ischemia" OR ischemia OR "Myocardial" OR "Ischemic Heart Disease" OR "Heart Disease" OR ischemic OR "Acute Coronary Syndrome" OR "Coronary Syndrome" OR "Acute Coronary" OR acs OR angina OR "Stable Angina" OR "Chronic Stable Angina" OR "Unstable Angina" OR "Coronary Disease" OR coronary OR "Coronary Stenosis" OR stenosis OR "Myocardial Infarction" OR infarction OR "Cardiovascular Stroke" OR "Cardiovascular Stroke" OR stroke OR cardiovascular OR "Myocardial Infarct" OR infarct OR "Heart Attack" OR "Heart Attack" OR mi OR "Non-ST Elevated Myocardial Infarction" OR "Non ST Elevated Myocardial Infarction" OR nstemi OR "Non-ST-Elevation Myocardial Infarction" OR "Non-ST-Elevation Myocardial" OR "Non-ST-Elevation" OR "Non ST Elevation Myocardial Infarction" OR "ST Elevation Myocardial Infarction" OR "ST Segment Elevation Myocardial Infarction" OR "ST Elevated Myocardial Infarction" OR stemi OR atherosclero* OR cardiovascular OR cardio* OR "Heart Disease" OR heart OR "Coronary Occlusion" OR stenosis OR "lipid profile" OR tc OR "Total cholesterol" OR tg OR triglycerides OR hdl OR ldl OR sbp OR "Systolic Blood Pressure" OR dbp OR "diastolic blood pressure" OR "blood pressure" ) OR TITLE-ABS-KEY ( "Coronary Artery Disease" OR "Artery Disease" OR "Coronary Arteriosclerosis" OR "Coronary Atherosclerosis" OR atherosclerosis OR cad OR "Myocardial Ischemia" OR ischemia OR "Myocardial" OR "Ischemic Heart Disease" OR "Heart Disease" OR ischemic OR "Acute Coronary Syndrome" OR "Coronary Syndrome" OR "Acute Coronary" OR acs OR angina OR "Stable Angina" OR "Chronic Stable Angina" OR "Unstable Angina" OR "Coronary Disease" OR coronary OR "Coronary Stenosis" OR stenosis OR "Myocardial Infarction" OR infarction OR "Cardiovascular Stroke" OR "Cardiovascular Stroke" OR stroke OR cardiovascular OR "Myocardial Infarct" OR infarct OR "Heart Attack" OR "Heart Attack" OR mi OR "Non-ST Elevated Myocardial Infarction" OR "Non ST Elevated Myocardial Infarction" OR nstemi OR "Non-ST-Elevation Myocardial Infarction" OR "Non-ST-Elevation Myocardial" OR "Non-ST-Elevation" OR "Non ST Elevation Myocardial Infarction" OR "ST Elevation Myocardial Infarction" OR "ST Segment Elevation Myocardial Infarction" OR "ST Elevated Myocardial Infarction" OR stemi OR atherosclero* OR cardiovascular OR cardio* OR "Heart Disease" OR heart OR "Coronary Occlusion" OR stenosis OR "lipid profile" OR tc OR "Total cholesterol" OR tg OR triglycerides OR hdl OR ldl OR sbp OR "Systolic Blood Pressure" OR dbp OR "diastolic blood pressure" OR "blood pressure" ) ) AND ( INDEXTERMS ( "randomized clinical trial" OR "Randomized controlled trial" OR "random allocation" OR "control group" OR randomized OR "clinical trial" OR random* OR trial OR blind OR "controlled trial" OR "controlled study" OR "randomized trial" ) OR TITLE-ABS-KEY ( "randomized clinical trial" OR "Randomized controlled trial" OR "random allocation" OR "control group" OR randomized OR "clinical trial" OR random* OR trial OR blind OR "controlled trial" OR "controlled study" OR "randomized trial" ) )
